# Lanosterol synthase deficiency promotes tumor progression by orchestrating PDL1‐dependent tumor immunosuppressive microenvironment

**DOI:** 10.1002/mco2.528

**Published:** 2024-04-10

**Authors:** Yuan Gao, Kun Zhao, Yulan Huang, Dapeng Zhang, Na Luo, Xiaoqing Peng, Feng Yang, Weidong Xiao, Meng Wang, Rongchen Shi, Hongming Miao

**Affiliations:** ^1^ Department of Pathophysiology College of High Altitude Military Medicine Third Military Medical University (Army Medical University) Chongqing China; ^2^ Department of Oncology Fuling Hospital Chongqing University Chongqing China; ^3^ Department of General Surgery Xinqiao Hospital Third Military Medical University (Army Medical University) Chongqing China; ^4^ Frontier Medical Training Brigade Third Military Medical University (Army Medical University) Xinjiang China; ^5^ Jinfeng Laboratory Chongqing China

**Keywords:** lanosterol synthase, programmed cell death ligand 1, tumor immunity, tumor microenvironment

## Abstract

Lipid metabolic reprogramming is closely related to tumor progression with the mechanism not fully elucidated. Here, we report the immune‐regulated role of lanosterol synthase (LSS), an essential enzyme in cholesterol synthesis. Database analysis and clinical sample experiments suggest that LSS was lowly expressed in colon and breast cancer tissues, which indicates poor prognosis. The biological activity of tumor cell lines and tumor progression in NOD scid gamma (NSG) mice were not affected after LSS knockdown, whereas LSS deficiency obviously aggravated tumor burden in fully immunized mice. Flow cytometry analysis showed that LSS knockdown significantly promoted the formation of tumor immunosuppressive microenvironment, characterized by the increase in M2 macrophages and polymorphonuclear myeloid‐derived suppressor cells (PMN‐MDSCs), as well as the decrease in anti‐tumoral T lymphocytes. With the inhibition of myeloid infiltration or loss function of T lymphocytes, the propulsive effect of LSS knockdown on tumor progression disappeared. Mechanistically, LSS knockdown increased programmed death ligand 1 (PDL1) protein stability by 2,3‐oxidosqualene (OS) binding to PDL1 protein. Anti‐PDL1 therapy abolished LSS deficiency‐induced immunosuppressive microenvironment and cancer progression. In conclusion, our results show that LSS deficiency promotes tumor progression by establishing an OS–PDL1 axis‐dependent immunosuppressive microenvironment, indicative of LSS or OS as a potential hallmark of response to immune checkpoint blockade.

## Graphical abstract

The low expression of LSS in tumor cells promotes intracellular OS accumulation, which then targets and stabilizes PDL1, leading to the decrease in tumor T lymphocyte activity and the increase in PMN‐MDSC and M2 like tumor associated macrophages (M2‐TAMs), and finally expedites tumor progression.

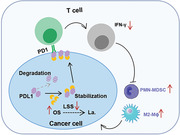

## 1 INTRODUCTION

Metabolic reprogramming within the tumor microenvironment (TME) plays a pivotal role in the initiation and progression of tumors.^1^, ^2^ The incessant proliferation of tumor cells necessitates an enormous demand for nutrients, resulting in persistent hypoxia, nutrient deprivation, and acidosis within the TME.^3^, ^4^ These conditions adversely impact the tumor immune cells, leading to immune suppression and evasion. Consequently, all cells within the TME must undergo metabolic reprogramming to acclimate and thrive in this unique milieu.^5^ Lipid metabolism has garnered significant attention in recent years due to its potential to serve as an energy source, as well as its ability to generate numerous intermediate metabolic and signaling molecules that impact tumor progression.^6^, ^7^

Lipid metabolism encompasses the metabolic processes of fats, phospholipids, and cholesterol. The reprogramming of lipid metabolism in tumor cells can have a direct or indirect impact on tumor progression.^7^ Our prior investigation revealed that ABHD5, a cofactor involved in the catabolism of triglycerides, was expressed at a low level in colon cancer cells, resulting in the accumulation of neutral fats and the malignant progression of tumors.^8^ Furthermore, silencing Agpat4 induces lysophosphatidic acid (LPA) released from tumor cells, which subsequently promotes p38/p65 phosphorylation through LPA receptors 1 and 3, resulting in M1 polarization and inhibition of tumor progression.^9^ Of course, cholesterol metabolism is also closely related to tumor cell biological activity and cancer progression.^10^, ^11^ And HMG‐CoA Reductase (HMGCR) can affect tumor immunity by regulating the expression of programmed death 1 (PD1) in regulatory T cells.^12^ However, the specific mechanisms of cholesterol metabolism and immune regulation in tumor cells are not fully understood.

In recent times, preclinical and clinical trials have yielded favorable outcomes for tumor immunotherapy.^13^ Notably, immune checkpoint blockade, encompassing cytotoxic T lymphocyte‐associated antigen 4 (CTLA4) and programmed death ligand 1 (PDL1), has emerged as the most efficacious approach.^13^, ^14^ Consequently, it is imperative to undertake further investigations into their intrinsic regulatory mechanisms to facilitate clinical treatment and mitigate the risk of toxic side effects by targeting CTLA4 and PDL1.^15^ In the context of lipid metabolism, recent findings suggest that gut microbial metabolites, specifically systemic short chain fatty acids, may impede the anti‐tumor effect of CTLA4 blockers.^16^ Yang et al. have demonstrated that PDL1 can facilitate tumor progression by preserving its own stability via palmitoylation, indicating a potential involvement of lipid metabolism in PDL1 expression.^17^ Therefore, it is imperative to investigate how tumor cells, particularly lipid metabolic reprogramming, regulate immune checkpoints for the prevention and treatment of tumors.

In this study, we conducted an analysis of cholesterol metabolism alterations between paracancerous and cancer tissues in colorectal cancer (CRC) and breast cancer (BRCA) through The Cancer Genome Atlas Program (TCGA) database analysis, revealing changes in various cholesterol synthesis‐related enzymes. In addition to HMGCR, the expression of lanosterol synthase (LSS), an important enzyme for cholesterol synthesis, was abnormally reduced in cancer tissues. Further experiments showed that LSS knockdown did not affect the biological activity of cancer cells, but could promote the formation of immunosuppressive microenvironment by binding 2,3‐oxidosqualene (OS) to PDL1 and stabilizing PDL1 protein, and ultimately accelerate the malignant progression of tumors.

## 2 RESULTS

### 2.1 Aberrant expression of LSS in human cancer tissues

**FIGURE 1:**
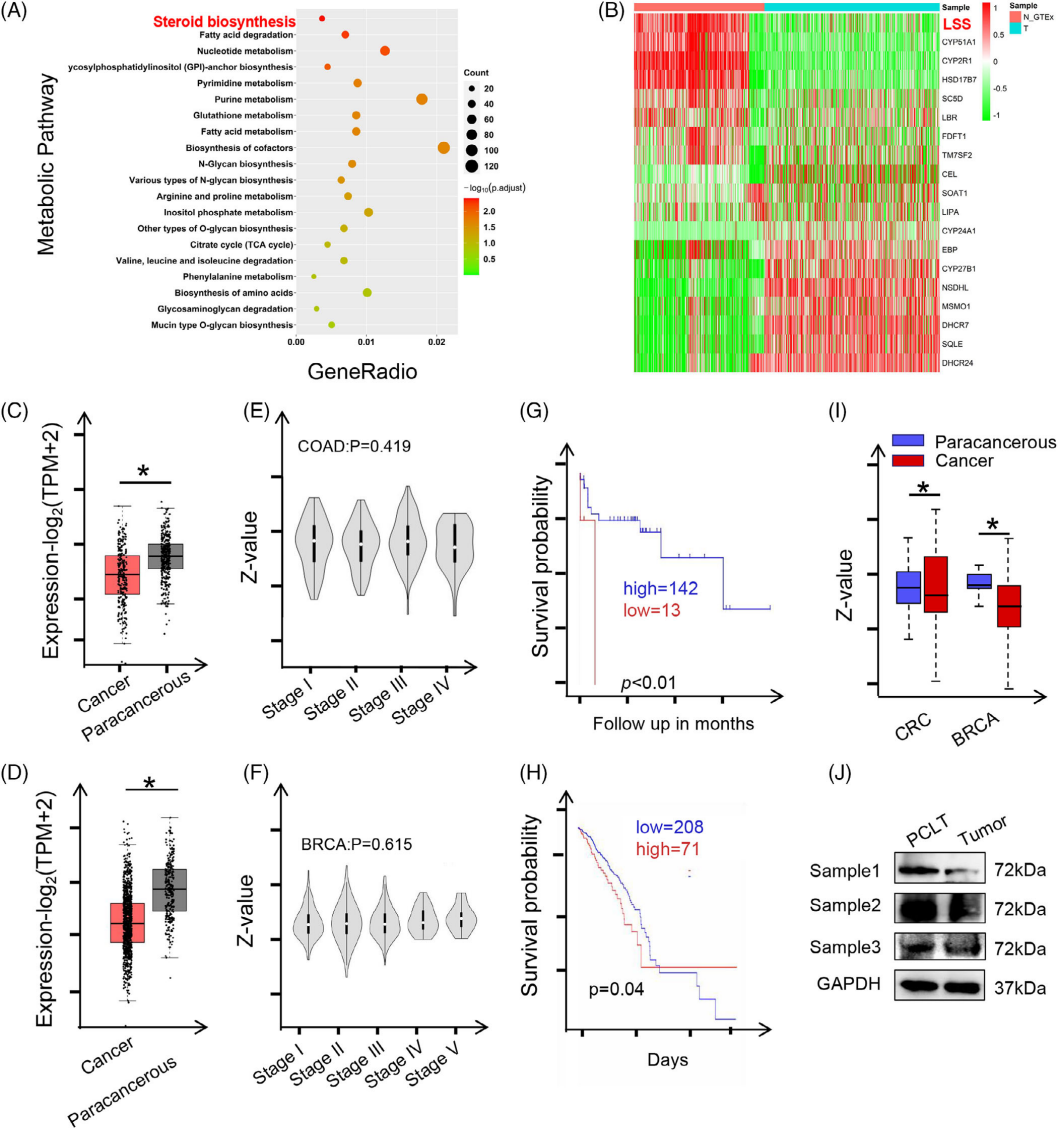
Aberrant expression of lanosterol synthase (LSS) in human cancer tissues. (A and B) The RNA sequencing results of adjacent tissues (Pc) and cancer tissues (Ca) of colorectal cancer (CRC) patients in TCGA database were analyzed. Gene Ontology (GO) pathway enrichment (A) and significantly differentially expressed genes in cholesterol metabolism (B) were performed as indicated. (C and D) The expression of LSS gene in cancer tissues of CRC or breast cancer (BRCA) patients. The sample size is shown in the figure. The relevant data were obtained from GEPIA database. (E and F) The expression of LSS gene in different stages of CRC or BRCA. The relevant data were obtained from GEPIA database. (G and H) The correlation between LSS gene expression and survival in CRC or BRCA patients. The sample size is shown in the figure. The relevant data were obtained from GEPIA database. The survival rates were analyzed by Kaplan–Meier survival analysis. (I) The expression of LSS protein in cancer tissues of CRC or BRCA patients. The relevant data were obtained from UALCAN database. (J) Protein levels of LSS in cancer and paracancerous tissues of CRC patients. All data represent the mean ± S.E.M. Except (D), which was analyzed with Gehan–Breslow–Wilcoxon test, the rest were analyzed with Student's *t*‐test (^*^ *p* < 0.05, ^**^ *p* < 0.01 and S.E.M., Standard Error of Mean; TCGA, The Cancer Genome Atlas Program).

To explore the effect of lipid metabolic reprogramming on tumor cells, we analyzed the change of lipid metabolism in colon and BRCA using TCGA database. Gene Ontology enrichment analysis showed significant changes in fatty acid metabolism and cholesterol metabolism (Figures 1A and S1A). Given that our previous studies have intensively explored the role of fatty acid metabolism in CRC, we next focused on cholesterol metabolism process. Further differential gene analysis revealed that LSS, an enzyme that catalyzes the production of lanosterol from OS,^18^ was abnormally down‐regulated in tumor cells (Figures 1B and S1B). Next, we used GEPIA database analysis to reconfirm the lower expression of LSS in CRC (Figure 1C) and BRCA (Figure 1D) tissues as compared to that in paracarcinoma tissues. However, the LSS expression was not associated with the tumor stage (Figure 1E,F). More importantly, low expression of LSS in cancer tissues of CRC and BRCA patients predicted poor prognosis (Figure 1G,H). In addition, UALCAN database analysis also suggested that LSS protein levels were significantly decreased in CRC and BRCA tissues (Figure 1I). These results were further verified in clinical samples with RNA (Figure S1C) and protein (Figure 1J) assays.

### 2.2 LSS knockdown did not affect the biological activity of tumor cell lines and tumor progression in NSG mice

**FIGURE 2:**
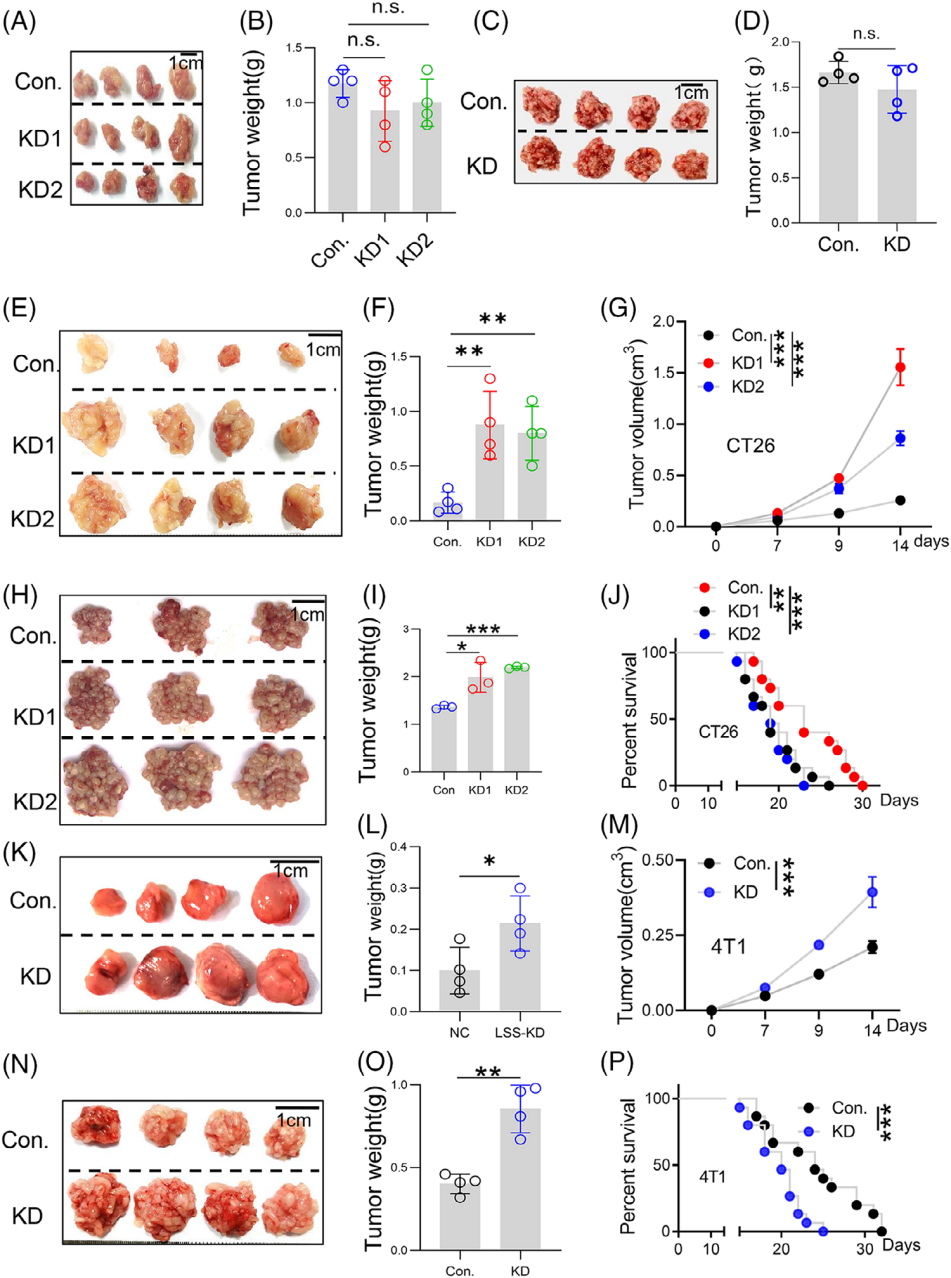
Lanosterol synthase (LSS) knockdown promoted tumor progression in fully immunized mice. (A and B) LSS‐NC (Con.) or LSS‐KD (KD) CT26 cells (5 × 10^6^ cells in 100 μL PBS) were subcutaneously injected into the groin of 6−8 weeks male NSG mice, and then tumor progression was observed 2 weeks later. (C and D) Con. or KD‐CT26 cells (5 × 10^6^ cells in 100 μL PBS) were intraperitoneally injected into the 6−8 weeks male NSG mice, and then tumor progression was observed 2 weeks later. (E and F) LSS knockdown in CT26 cells inhibited subcutaneous tumor progression. Con. or KD‐CT26 cells (5 × 10^6^ cells in 100 μL PBS) were subcutaneously injected into the groin of 6−8 weeks male BABL/C mice, and then tumor progression was observed 2 weeks later (*n* = 3). (G) Dynamic observation of mouse tumor volume in (E). (H and I) LSS knockdown in CT26 cells inhibited colorectal cancer (CRC)‐PC progression. Six to eight weeks male BABL/C mice were intraperitoneally injected with CT26 cells (Con., KD1, or KD2), and then tumor progression was observed 2 weeks later (*n* = 3). (J) The mouse CRC‐PC model was established as in (D), and the survival time of the BABL/C mice was observed (*n* = 3). (K and L) LSS knockdown in 4T1 cells inhibited subcutaneous tumor progression in C57 mice. Method such as (A) (*n* = 3). (M) Dynamic observation of mouse tumor volume in (K) (*n* = 3). (N and O) LSS knockdown in 4T1 cells inhibited CRC‐PC progression. Six to eight weeks male C57 mice were intraperitoneally injected with 4T1 cells (Con. or KD), and then tumor progression was observed 2 weeks later (*n* = 3). (P) The mouse CRC‐PC model was established as in (H), and the survival time of the mice was observed (*n* = 3). All data represent the mean ± S.E.M. Except (F) and (L), which were analyzed with Gehan–Breslow–Wilcoxon test, the rest were analyzed with Student's *t*‐test (^*^ *p* < 0.05, ^**^ *p* < 0.01, ^***^ *p* < 0.005, and KD, Knock‐down; n.s.: not significant; NSG, NOD scid gamma; PBS: Phosphate buffer saline; S.E.M., Standard Error of Mean).

To further explore the role of LSS in tumor cells, lentivirus‐mediated shRNAs were used to knockdown LSS in tumor cells (Figure S2A–C), and the biological activity of tumor cells was observed. As shown in Figure S2D,G, LSS knockdown did not affect the cell proliferation activity. Further experiments also showed that there was no significant change in cell apoptosis (Figure S2E,H) or cell cycle (Figure S2F,I) after LSS knockdown. Similar results were observed in BRCA 4T1 cells (Figure S2J–L). And LSS knockdown also did not affect the migration (Figure S2M) and invasion (Figure S2N) of CT26 cells. Importantly, LSS knockdown did not affect the subcutaneous (SC) tumor growth (Figure 2A,B) and CRC‐PC (Figure 2C,D) of CT26 cells in NOD scid gamma (NSG) mice.

### 2.3 LSS knockdown promoted tumor progression in fully immunized mice

Combined with the prognostic analysis of the database and tumor cell biological activity results, we speculate that the role of LSS in tumor is immunologically related. Further experiments showed that knockdown of LSS significantly promoted tumor growth in SC tumor model of CT26 cells (Figure 2E–G). In addition, LSS knockdown also exacerbated the CRC‐PC progression (Figure 2H,I) and shortened the mice survival time (Figure 2J). Identical results were found in 4T1 cells (Figure 2K–P). Taken together, the above results suggest that the effect of LSS knockdown on tumor progression might be related to the immune modulation of the TME.

To explore the relationship between LSS and tumor immunity, we preliminarily used the TIMER database to predict the correlation of its expression with immune infiltration level in CRC and BRCA. Surprisingly, there was no significant correlation between LSS expression and tumor immune infiltration (Figure S3A,B). However, Somatic copy number changes (SCNA) algorithm was used to further explore the relationship between the somatic copy number of LSS and the infiltration of tumor immune cells, which showed that LSS significantly affected the infiltration of a variety of tumor‐associated immune cells (Figure S3C).

### 2.4 LSS knockdown promotes tumor progression depending on myeloid cells

**FIGURE 3:**
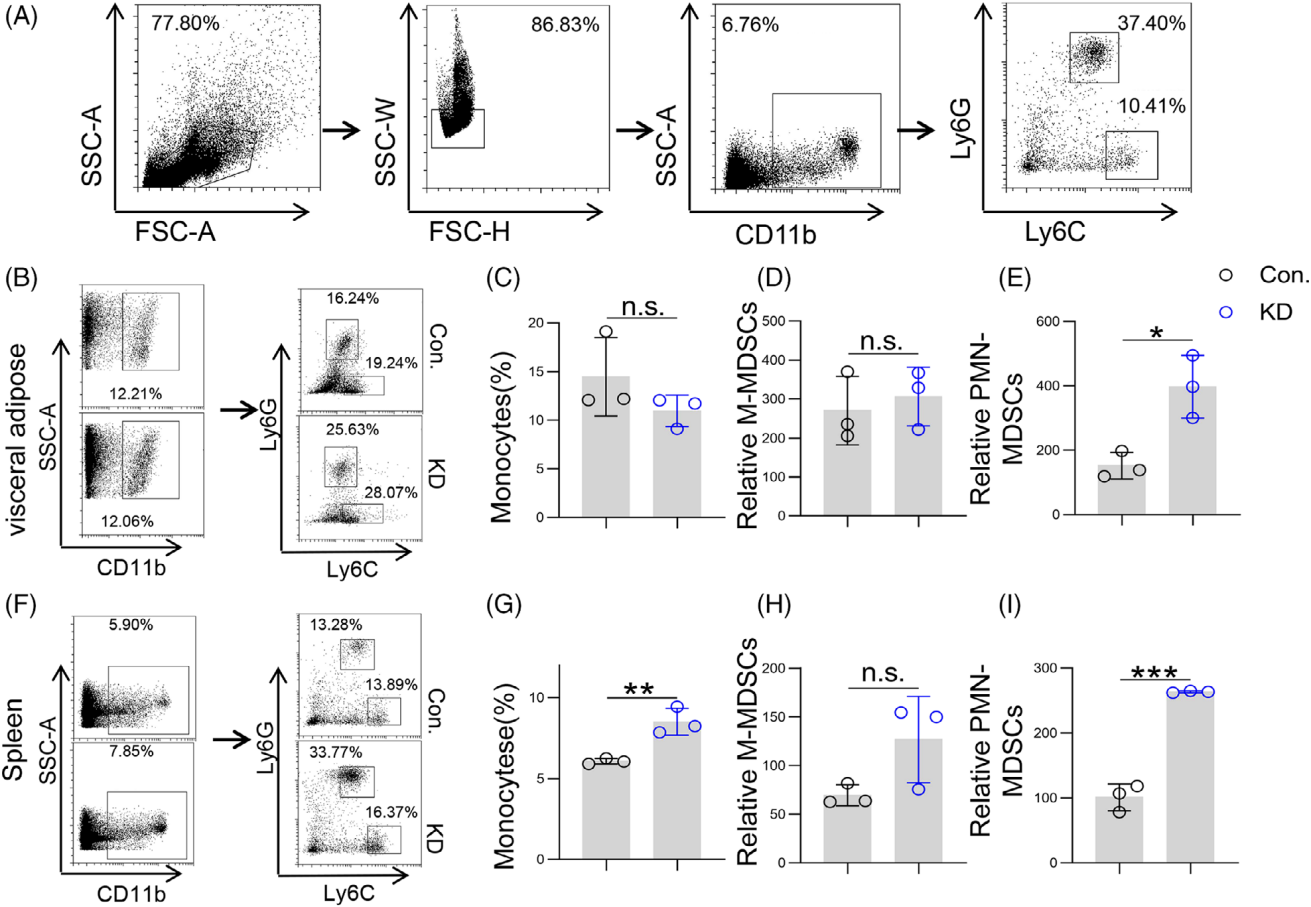
Lanosterol synthase (LSS) knockdown increased the proportion of polymorphonuclear myeloid‐derived suppressor cells (PMN‐MDSCs). (A) Strategies for FACS analysis of tumor and splenic MDSC. After removal of particles, cell debris, and adherent cells, monocytes were then assessed as CD11b^+^, PMN‐MDSC were assessed as CD11b^+^Ly6G^+^Ly6C^low^, and monocyte‐type MDSC (M‐MDSC) were assessed as CD11b^+^Ly6G^−^Ly6C^hi^. (B) Percentages or number of MDSC and their subtypes in visceral adipose tissue. Six to eight weeks male BABL/C mice were intraperitoneally injected with CT26 cells (Con. or KD), and then the percentages of MDSC and their subtypes in visceral adipose tissue were analyzed by flow cytometry on day 5. Each sample was made by mixing visceral adipose tissue from two to three mice. Representative result was shown (*n* = 3). (C–E) The proportion of visceral adipose monocytes (C) and the relative number of PMN‐MDSC (D) and M‐MDSC (E) as described in (B) (*n* = 3). (F) Infiltration of MDSC and their subtypes in spleen. Method as described in (B) (*n* = 3). (G–I) The proportion of monocytes (G) and the relative number of PMN‐MDSC (H) and M‐MDSC (I). Method as described in (F). All data represent the mean ± S.E.M. (^*^ *p* < 0.05, ^**^ *p* < 0.01, and n.s., not significant; S.E.M., Standard Error of Mean; Student's *t*‐test).

To further clarify whether LSS knockdown could regulate tumor immunity, we examined the changes of several tumor‐associated immune cells. Myeloid cells play an important role in the whole process of tumor development and drug resistance. Myeloid‐derived suppressor cells (MDSCs), which are composed of immature myeloid cells, are a representative heterogeneous group in the TME and also show strong immunosuppressive activity, including two subgroups monocyte‐type MDSC (M‐MDSC) and polymorphonuclear MDSC (PMN‐MDSC).^19^ To further explore the effect of LSS knockdown on tumor immune microenvironment, we also detected the changes of MDSCs in TME by flow cytometry. We found that although LSS knockdown did not affect the infiltration of monocytes or M‐MDSCs in visceral adipose tissue (Figure 3A–D), it observably increased the number of PMN‐MDSCs (Figure 3E). Given that systemic immunity is also critical for tumor progression,^20^ we also found that LSS knockdown increased the percentage of monocytes (Figure 3F,G) and the number of PMN‐MDSC (Figure 3I), but did not affect M‐MDSC (Figure 3H) in the spleen, suggesting that PMN‐MDSCs are also involved in the anti‐tumor effect of LSS.

**FIGURE 4:**
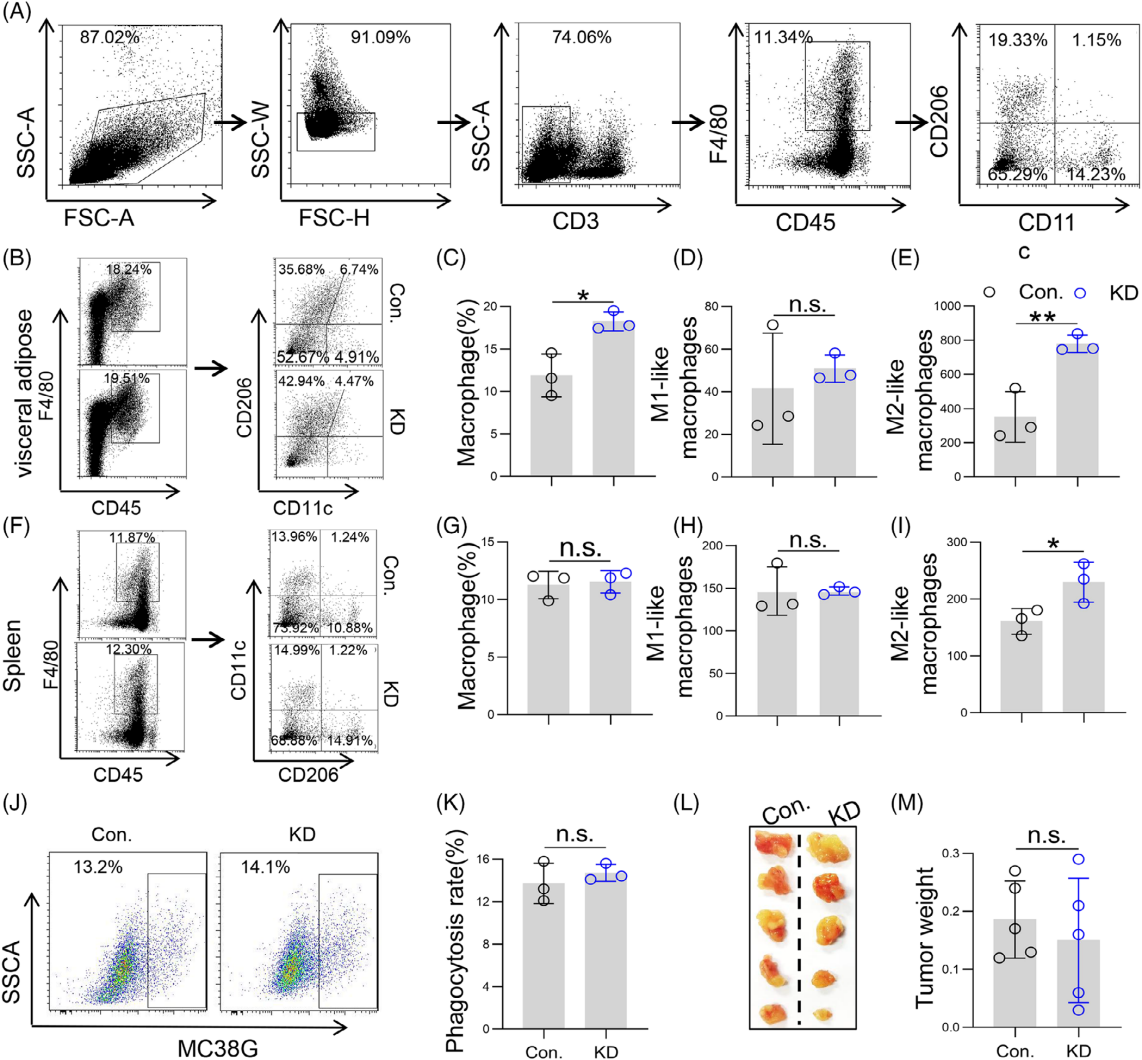
Lanosterol synthase (LSS) knockdown promoted tumor progression depending on macrophages. (A) Strategies for FACS analysis of tumor and splenic macrophages. After removal of particles, cell debris, and adherent cells, macrophages were then assessed as CD902^−^ CD45^+^F4/80^+^, M1‐like macrophages were assessed as CD902^−^ CD45^+^F4/80^+^CD11c^+^CD206^−^, and M2‐like macrophages were assessed as CD902^−^ CD45^+^F4/80^+^CD11c^−^CD206^+^. (B) Percentages or number of macrophages and their subtypes in visceral adipose tissue. Six to eight weeks male BABL/C mice were intraperitoneally injected with CT26 cells (Con. or KD), and then the percentages of macrophages and their subtypes in visceral adipose tissue were analyzed by flow cytometry on day 5. Each sample was made by mixing visceral adipose tissue from two to three mice. Representative result was shown (*n* = 3). (C–E) The proportion of macrophages (C) and the relative number of M1‐like macrophages (D) and M2‐like macrophages (E) as described in (B). (F) Infiltration of macrophages and their subtypes in spleen. Method as described in (B) (*n* = 3). (G–I) The proportion of splenic macrophages (G) and the relative number of M1‐like macrophages (H) and M2‐like macrophages (I). Method such as (F). (J and K) Phagocytic activity of macrophages. Mouse bone marrow‐derived macrophages (BMDMs) were extracted and co‐incubated with CT26 cells of Con. or KD at a ratio of 1:5 for 4 h. After collecting the cells for staining, the phagocytosis rate of macrophages was detected by flow cytometry. (L and M) Con. or KD‐4T1 cells (5 × 10^6^ cells in 100 µL PBS) were subcutaneously injected into the groin of 6−8 weeks male myeloid^KO^ mice, and then tumor progression was observed 2 weeks later (*n* = 3). All data represent the mean ± S.E.M. (^*^ *p* < 0.05, ^**^ *p* < 0.01, and KD, Knock‐down; n.s.: not significant; PBS, Phosphate buffer saline; S.E.M., Standard Error of Mean; Student's *t*‐test).

As the largest group of myeloid cells in the TME, macrophages are also closely related to tumorigenesis, tumor development, metastasis, and drug resistance.^21^ Therefore, we first detected the polarization of TAMs in the tumor by flow cytometry. Results showed that LSS knockdown increased the proportion of macrophages (Figure 4A–D) and the number of M2‐like macrophages in visceral adipose tissue (Figure 4E). In addition, we also detected and found that LSS knockdown significantly promoted M2 polarization of splenic macrophages, although it did not affect the percentage of total splenic macrophages and M1‐like macrophages (Figure 4F–I). In addition to polarization, macrophages could also directly phagocytose tumor cells for anti‐tumor.^22^ Further experiments revealed that LSS knockdown did not affect macrophage phagocytosis (Figure 4J,K). Notably, the tumor‐promoting effect mediated by LSS knockdown was abolished in myeloid^KO^ mice (Figure 4L,M), which suggested that macrophages and MDSCs may mediate the anti‐tumor effect of LSS.

### 2.5 LSS knockdown inhibited T lymphocyte activity and promoted tumor progression

**FIGURE 5:**
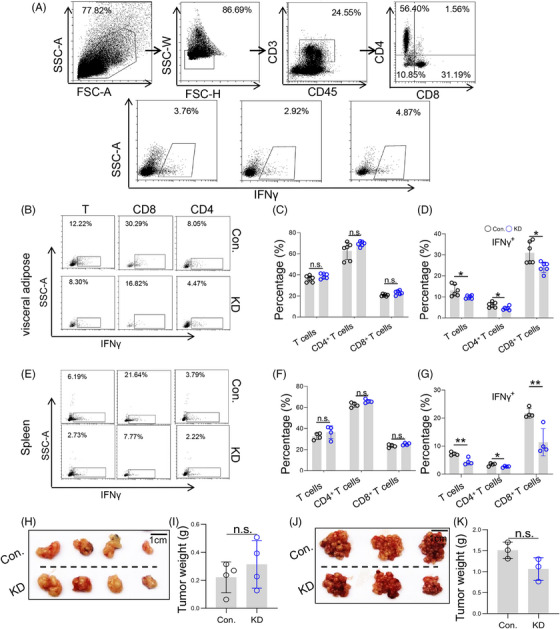
Lanosterol synthase (LSS) knockdown inhibited T lymphocyte activity and promoted tumor progression. (A) Strategies for FACS analysis of tumor and splenic T lymphocytes. After removal of particles, cell debris, and adherent cells, T cells were assessed as CD45^+^CD3^+^, CD8^+^ T cells were assessed as CD45^+^CD3^+^CD8^+^CD4^−^, CD4^+^ T cells were assessed as CD45^+^CD3^+^CD8^−^CD4^+^, IFNγ^+^ T cells were assessed as CD45^+^CD3^+^ IFNγ^+^, IFNγ^+^ CD8^+^ T cells were assessed as CD45^+^CD3^+^CD8^+^CD4^−^IFNγ^+^, and IFNγ^+^CD4^+^ T cells were assessed as CD45^+^CD3^+^CD8^−^CD4^+^ IFNγ^+^. (B) Percentage of T lymphocytes and their subtypes in visceral adipose tissue. Six to eight weeks male BABL/C mice were intraperitoneally injected with CT26 cells (Con. or KD), and then the percentages of T lymphocytes in visceral adipose tissue were analyzed by flow cytometry on day 5. Each sample was made by mixing visceral adipose tissue from two to three mice. Representative result was shown (*n* = 3). (C and D) The proportion of T lymphocytes, CD8^+^ T cells, CD4^+^ T cells, IFNγ^+^ T, IFNγ^+^ CD8^+^ T, and IFNγ^+^CD4^+^ T cells as described in (B) (*n* = 3). (E) Percentage of T lymphocytes and their subtypes in spleen. Method as described above in (B) (*n* = 3). (F and G) The proportion of splenic T lymphocytes, CD8^+^ T cells, CD4^+^ T cells, IFNγ^+^ T, IFNγ^+^ CD8^+^ T, and IFNγ^+^CD4^+^ T cells. Method as described above in (E). (H and I) Con. or KD‐CT26 cells (5 × 10^6^ cells in 100 µL PBS) were subcutaneously injected into the groin of 6−8 weeks male nude mice, and then tumor progression was observed 2 weeks later (*n* = 3). (J and K) Con. or KD‐CT26 cells (5 × 10^6^ cells in 100 µL PBS) were intraperitoneally injected into the 6−8 weeks male nude mice, and then tumor progression was observed 2 weeks later. All data represent the mean ± S.E.M. (^*^ *p* < 0.05, ^**^ *p* < 0.01, and n.s., not significant; PBS, Phosphate buffer saline; S.E.M., Standard Error of Mean; Student's *t*‐test).

Compared with tumor innate immunity, adaptive immunity plays a more important role in tumor immune regulation, especially tumor‐associated T lymphocytes.^23^ To further explore whether T lymphocytes mediate the anti‐tumor effect of LSS, we continued to examine the changes of tumor T lymphocytes, including CD8^+^ and CD4^+^ T lymphocytes, by flow cytometry (Figure 5A). Unexpectedly, LSS knockdown did not affect the percentage of total T, CD8^+^ T, and CD4^+^ T cells in visceral adipose tissue (Figure 5B,C). And LSS knockdown did not affect the cell proliferation of T lymphocytes (Figure S4A). Compared with the changes in number, the activity of tumor T lymphocytes can better reflect their anti‐tumor function. Interferon‐gamma (IFNγ) is a well‐recognized T lymphocyte anti‐tumor cytokine, which significantly inhibits tumor growth and metastasis, and can better represent T lymphocyte activity.^24^ Therefore, we went on to examine the expression of IFNγ in visceral adipose T lymphocytes and found that LSS knockdown markedly reduced the expression of IFNγ in T lymphocytes, including CD8^+^ T and CD4^+^ T cells (Figure 5D). Similarly, the number of splenic T lymphocytes did not change but dramatically inhibited their IFNγ expression after LSS knockdown (Figure 5E–G). Importantly, LSS knockdown failed to promote tumor progression in nude mice, including SC tumor models (Figure 5H,I) and CRC‐PC models (Figure 5J,K). Altogether, the above results suggest that LSS knockdown might promote tumor progression by inhibiting T lymphocyte activity.

### 2.6 OS targeting and stabilizing PDL1 orchestrates an immunosuppressive microenvironment to promote tumor progression

The aforementioned results indicated that LSS knockdown signally promotes the formation of tumor immunosuppressive microenvironment and tumor progression. Since LSS is an important enzyme for cholesterol synthesis, to further explore the underlying mechanism of LSS‐mediated tumor suppression, we first examined the changes in cholesterol content. However, although LSS knockdown reduced cholesterol content in cells and supernatant (Figure S5A,B), previous studies have found that cholesterol deficiency inhibits the anti‐tumor response of immune cells, including T lymphocytes^25^ and macrophages.^26^ We also found no significant difference in the expression of anti‐inflammatory cytokines in macrophages (Figure S5C) and IFNγ in T lymphocytes (Figure S5D) after treatment with LSS knockdown tumor conditioned medium (CM), suggesting that cell‐to‐cell interaction might mediate the above tumor‐promoting effect.

**FIGURE 6:**
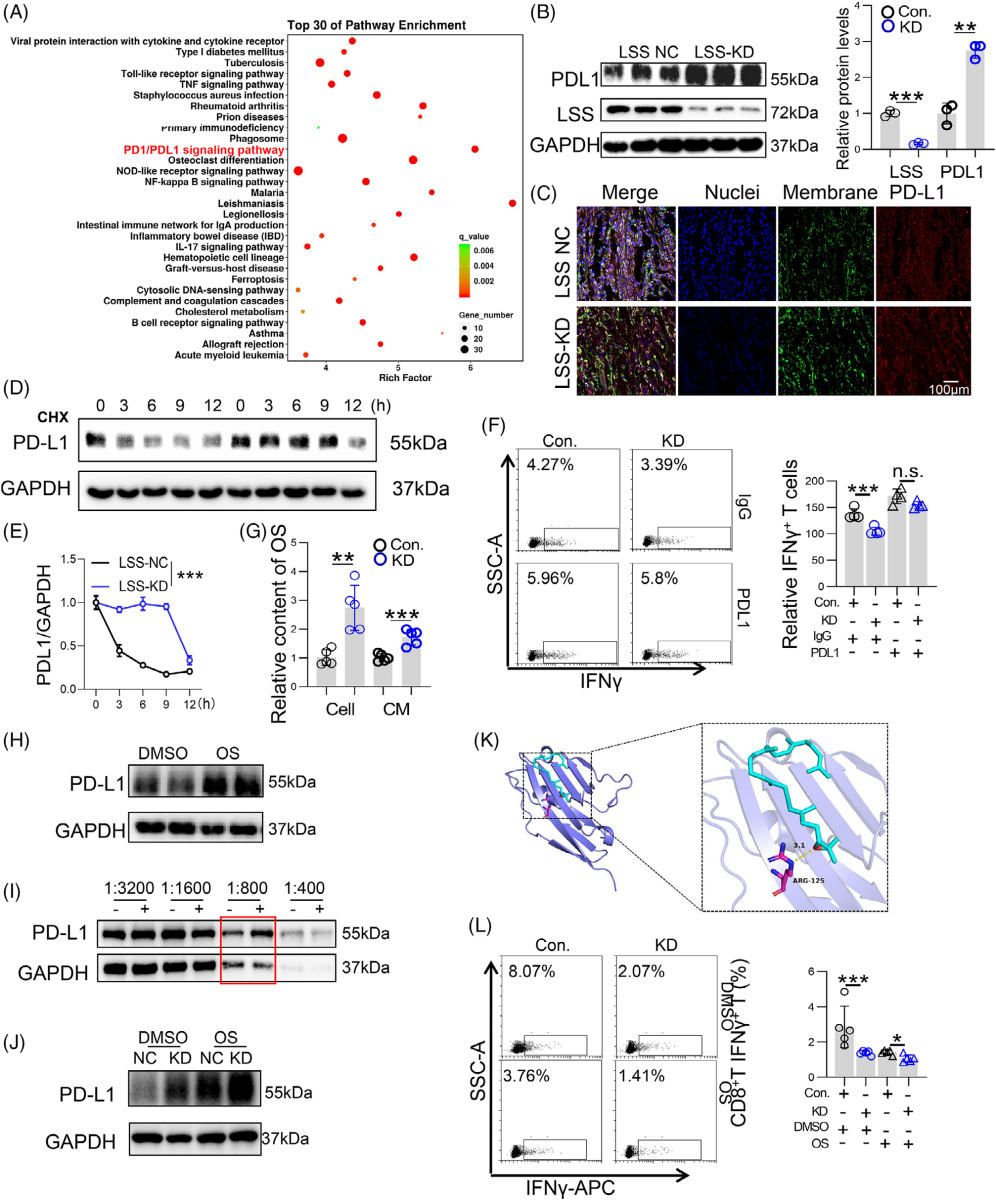
2,3‐Oxidosqualene (OS) targeting and stabilizing programmed death ligand 1 (PDL1) constructed an immunosuppressive microenvironment to promote tumor progression. (A) The PD1/PDL1 signaling pathway were highly enriched. Lanosterol synthase (LSS)‐KD or LSS‐NC CT26 cells were analyzed by RNA sequencing, and significantly altered genes were analyzed by Kyoto Encyclopedia of Genes and Genomes (KEGG) pathway enrichment analysist. (B) The protein level of PDL1. Immunoblotting assays were used to measure PDL1 protein levels in LSS‐NC or LSS‐KD CT26 cells (*n* = 3). (C) The PD‐L1 expression levels at the cell plasma membrane (*n* = 3). (D and E) Measure of PDL1 protein stability. LSS‐NC or LSS‐KD CT26 cells were treated with CHX for 0, 3, 6, 9, and 12 h, and PDL1 protein levels were detected by Western blot (*n* = 3). (F) IFNγ expression in T lymphocytes. LSS‐NC or LSS‐KD CT26 cells were preincubated with immunoglobulin G (IgG) or PDL1 neutralizing antibody for 4 h, and then splenic leukocytes were added for 6 h. The expression of IFNγ in T lymphocytes was detected by flow cytometry (*n* = 3). (G) Intracellular (cell) or supernatant (conditioned medium) concentrations of OS in Con.‐ or KD‐transfected CT26 cells. (H) The protein level of PDL1. Immunoblotting assays were used to measure PDL1 protein levels in CT26 cells after 36 h of DMSO or OS (80 µM) treatment (*n* = 3). (I) The protein level of PDL1. Drug affinity responsive target stability (DARTS) experiments are performed as described in Section 2. (J) The protein level of PDL1. Immunoblotting assays were used to measure PDL1 protein levels in Con. or KD‐CT26 cells after 36 h of DMSO or OS (80 µM) treatment (*n* = 3). (K) Molecular docking of OS and PDL1 protein was performed to obtain the molecular–protein interaction map. The PD1L1 protein is represented as a cartoon model in dark blue, the ligand is shown as a cyan stick model, and their binding sites are shown as a magenta stick structure. Nonpolar hydrogen atoms are omitted. Hydrogen bonding, ionic interactions, and hydrophobic interactions are depicted as yellow, magnetic, and green dashed lines, respectively. (L) IFNγ expression in T lymphocytes. Splenic leukocytes were preincubated with DMSO or OS for 4 h, and then Con. or KD‐CT26 cells were added for 6 h. The expression of IFNγ in T lymphocytes was detected by flow cytometry (*n* = 3). All data represent the mean ± S.E.M. (^**^ *p* < 0.01, ^***^ *p* < 0.005, and CHX, Cycloheximide; DMSO, Dimethyl sulfoxide; KD, Knock‐down; n.s. not significant; S.E.M., Standard Error of Mean; Student's *t*‐test).

As expected, we found that direct coculture of LSS knockdown tumor cells with splenic stromal cells significantly impaired the level of IFNγ in splenic T lymphocytes (Figure S5E). Therefore, RNA‐sequencing assays were used to further explore the signaling differences between the control and LSS‐silenced CT26 cells. We found that multiple immune‐related signals were obviously altered after LSS knockdown, especially PD1/PDL1 signaling pathway (Figure 6A), which is the immune checkpoint signaling pathway that negatively regulates T lymphocyte activation. Next, we verified that LSS knockdown dramatically increased PDL1 protein levels (Figure 6B), especially at the cell membrane (Figure 6C), but did not affect its mRNA levels in tumor cells (Figure S5F). We also demonstrated that LSS knockdown enhanced PDL1 protein stability (Figure 6D,E). Notably, we found that PDL1 inhibitor treatment abolished the effect of LSS knockdown on attenuating T lymphocyte activity (Figure 6F), suggesting that the increase in PDL1 protein level mediates the tumor‐promoting effect.

Given that LSS mainly promotes the conversion of OS to lanosterol, to further explore how LSS knockdown regulates the protein stability of PDL1, we first detected and found that LSS knockdown caused a significant increase in OS in intracellular and CM (Figure 6G), whereas lanosterol was only slightly reduced in cells (Figure S5G). And we found that OS (Figure 6H), but not lanosterol (Figure S5H), significantly increased PDL1 protein levels in CT26 cells. More importantly, drug affinity responsive target stability (DARTS) assay showed that OS could directly bind to PDL1 (Figure 6I) and eliminate the PDL1 protein increase caused by LSS knockdown (Figure 6J). However, lanosterol failed to bind to the PDL1 protein in DARTS assay (Figure S5I). In addition, molecular docking of OS and mouse PDL1 protein using Autodock Vina (1.2.0) revealed that ARG125 of PD1L1 was a possible binding site of OS (Figure 6K). Finally, treatment of OS obviously abolished KD‐mediated reduction in IFNγ secretion of splenic T lymphocytes (Figure 6L). Taken together, the above results suggest that LSS knockdown might increase intracellular OS, which in turn targets PDL1 to inhibit T lymphocyte activity and promote tumor progression.

### 2.7 PDL1 neutralizing antibody blocked the immunosuppression and tumor promotion mediated by LSS knockdown

**FIGURE 7:**
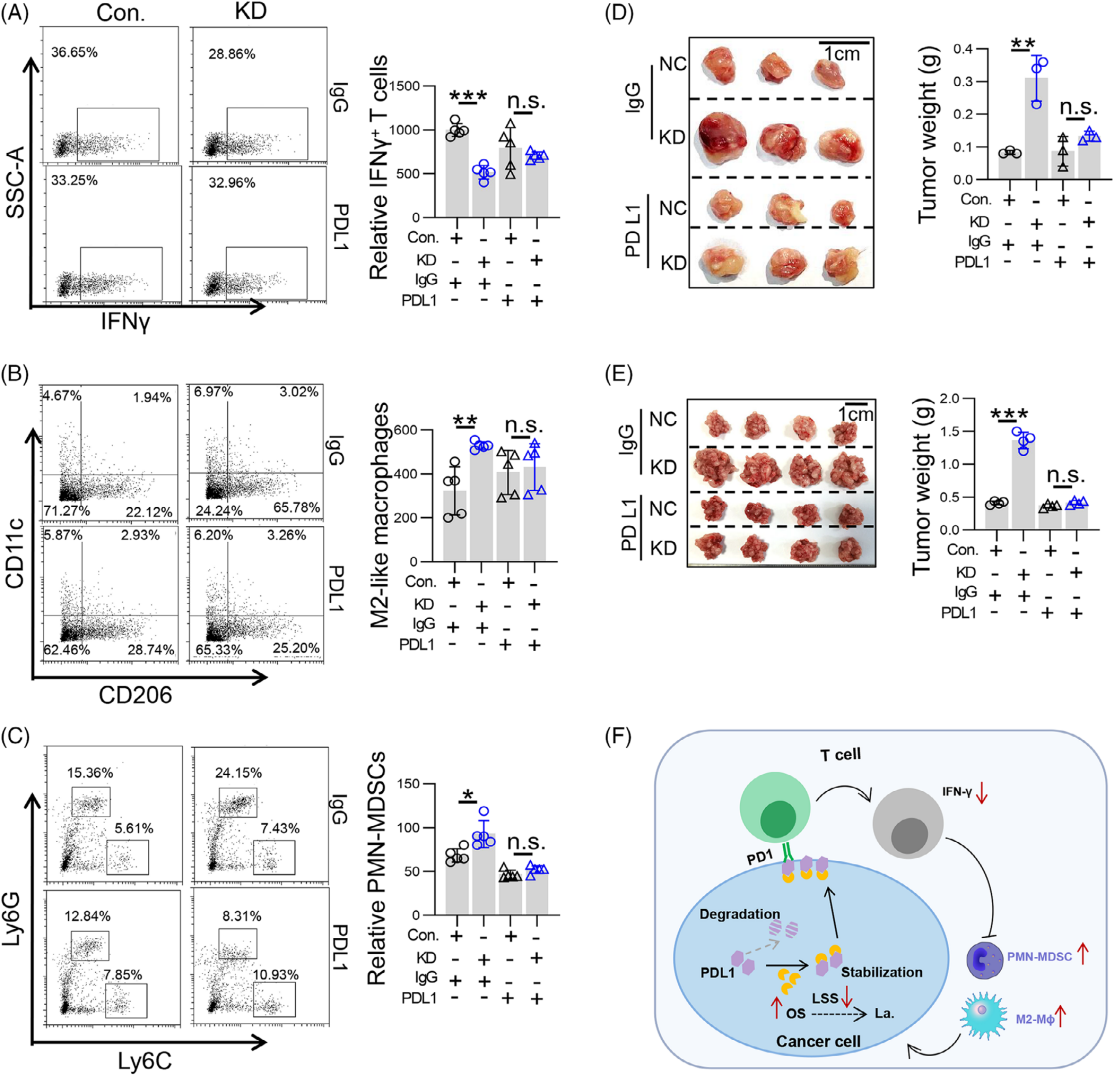
Lanosterol synthase (LSS) knockdown promoted tumor progression through the formation of programmed death ligand 1 (PDL1)‐mediated immunosuppressive microenvironment. (A) Relative number of IFNγ^+^ T lymphocytes. Six to eight weeks male BABL/C mice were intraperitoneally injected with CT26 cells (Con. or KD) and were treated with PDL1 neutralizing antibody on days 0, 2, and 4. Then, the percentages of T lymphocytes in visceral adipose tissue were analyzed by flow cytometry on day 5. Each sample was made by mixing visceral adipose tissue from two to three mice. Representative result was shown (*n* = 3). (B) Relative number of M2‐like macrophages. Methods such as (A) (*n* = 3). (C) Relative number of polymorphonuclear myeloid‐derived suppressor cells (PMN‐MDSCs). Methods such as (A) (*n* = 3). (D) Six to eight weeks male BABL/C mice were injected subcutaneously with CT26 cells (Con. or KD), and then PDL1 neutralizing antibody or immunoglobulin G (IgG) was injected on days 0, 2, and 4. Finally, mice were sacrificed on day 14 to observe tumor progression (*n* = 3). (E) Six to eight weeks male BABL/C mice were intraperitoneally injected with CT26 cells (Con. or KD), and then PDL1 neutralizing antibody or IgG was injected on days 0, 2, and 4. Finally, mice were sacrificed on day 14 to observe tumor progression (*n* = 3). (F) Graphical abstract. The low expression of LSS in tumor cells promotes intracellular 2,3‐oxidosqualene (OS) accumulation, which then targets and stabilizes PDL1, leading to the decrease in tumor T lymphocyte activity and the increase in PMN‐MDSC and M2‐TAMs, and finally expedites tumor progression. All data represent the mean ± S.E.M. (^*^ *p* < 0.05, ^**^ *p* < 0.01, and M2‐TAMs: M2 like tumor associated macrophages; n.s, not significant; S.E.M., Standard Error of Mean; Student's *t*‐test).

We next further validated the role of PDL1 in the aforementioned mechanisms in vivo. We found that PDL1 neutralizing antibodies inhibited the effect of diminished T lymphocyte activity induced by LSS knockdown (Figure 7A). Similarly, the increased numbers of M2‐like macrophages and PMN‐MDSCs were abolished after treatment with PDL1 neutralizing antibody (Figure 7B,C). Finally, we performed PDL1 neutralizing antibody treatment and found that the tumor‐promoting effect mediated by LSS knockdown disappeared, including SC tumor model (Figure 7D) and CRC‐PC model (Figure 7E). Taken together, these results suggest that the OS/PDL1/PD1 signaling axis mediates the tumor promotion caused by LSS knockdown.

## 3 DISCUSSION

Our results suggest a novel relationship between cholesterol metabolism and the immune checkpoint PDL1 in cancer cells to the tumor growth. Initially, we observed that the gene LSS, which is crucial for cholesterol metabolism, was down‐regulated in both CRC and BRCA, and was linked to unfavorable prognosis. The diminished expression of LSS in tumor cells may facilitate tumor immunosuppression and progression via OS–PDL1 axis, thereby indicating that the level of OS or LSS could serve as a potential biomarker for the clinical use of PD1/PDL1 inhibitors (Figure 7F).

Targeted inhibition of the active cholesterol metabolism of tumor cells has been demonstrated to be a feasible anti‐tumor approach.^10^ Prior research has demonstrated that certain enzymes, such as HMGCR and squalene synthase, which are involved in cholesterol synthesis, exhibit notable anti‐tumor or anti‐proliferative properties.^10^, ^27^ Notably, HMGCR inhibitors, such as statins, have been shown to significantly reduce mortality rates and extend survival periods in cancer patients, rendering them the most commonly employed drugs in clinical investigations targeting cholesterol metabolism in cancer patients.^28^ Nevertheless, our current investigation reveals that LSS, a critical enzyme in the latter stages of cholesterol synthesis, is down‐regulated in CRC and BRCA cells and significantly promoted tumor progression and shortened patient survival. Furthermore, the reduction of cellular cholesterol content was also observed with LSS knockdown, indicating different enzymes or derivatives of cholesterol synthesis may have different tumor regulatory effects. Notably, we found that the tumor‐promoting effect of LSS knockdown might be mediated by OS, which could directly target PDL1 to enhance its protein stability. The above results suggest the role of cholesterol metabolic intermediates in immunotherapy and the need to further elucidate the other potential binding metabolites of PDL1 and its mechanism of tumor regulation.

PDL1 is a critical factor in the formation of an immunosuppressive microenvironment within tumors, thereby facilitating immune evasion.^29^ Prior investigations have demonstrated that PDL1 binding to T cell PD1 results in the activation of protein tyrosine phosphatases, including SHP2, and the promotion of transcription factor expression, such as BATF, which collectively antagonize T‐cell receptors (TCR‐) and CD28‐mediated signaling. This ultimately leads to the functional suppression of effector T cells and T‐cell exhaustion.^30^ Furthermore, Weissman and coworkers have reported that macrophage PD1 expression inhibits phagocytosis and tumor immunity.^31^ Despite the absence of observed changes in macrophage phagocytosis, a noteworthy elevation in M2‐like macrophages was detected, potentially linked to reduced T‐cell function. Interestingly, the administration of a PDL1 neutralizing antibody did not exhibit any anti‐tumor properties on normal CT26 cells xenografts, despite significantly impede tumor growth following LSS knockdown. We hypothesized that this might be related to the weak expression of PDL1 in CT26 cells under normal conditions, resulting in the difficulty of neutralizing antibodies to exert effective anti‐tumor function. Once the level of PDL1 protein in cells is increased, such as after LSS knockdown, the effect of neutralizing antibodies is also highlighted. This outcome suggests that LSS could serve as a biomarker for cancer immunotherapy, whereby tumors with low LSS expression may be more amenable to PDL1/PD1 inhibitor treatment.

In conclusion, we are the first to show that LSS in tumor cells may suppress tumor growth through inhibiting OS/PDL1 axis‐mediated immune escape. Our findings are expected to provide new ideas for the treatment of PDL1/PD1‐targeted therapy resistance, and provide theoretical basis for the development of intervention strategies based on tumor immune microenvironment.

## 4 MATERIALS AND METHODS

### 4.1 Database analysis

The data of BRCA and CRC patients were obtained from TCGA database (portal.gdc.cancer.gov/). After downloading the data, the conventional script was run to analyze differential signaling pathways and genes. The rest are all online database analysis, including GEPIA (http://gepia.cancer‐pku.cn/index.html) and UALCAN (https://ualcan.path.uab.edu/)

### 4.2 Cell culture

There are two mouse tumor cell lines preserved in our laboratory, CT26 (from colon cancer) and 4T1 (from BRCA). All cells have been identified and mycoplasma tested. CT26 cell lines were cultured in Dulbecco's modified Eagle culture medium with 10% hyclone high‐quality fetal bovine serum (FBS) and 5% penicillin streptomycin double antibody at 37°C in a humid environment of 5% CO_2_. Other cell lines were using normal FBS, and other conditions were the same as those of CT26 cell lines.

### 4.3 Mice

The Animal Care and Use Committee of the Army Medical University (AMU) has reviewed and approved the protocol for the use of animals in this study. Mice were handled and cared for in strict accordance with the ethical guidelines and conditions set by AMU's Animal Care and Use Committee. Wild‐type C57BL/6 and BALB/c mice, as well as BALB/c nude and NSG mice, were provided by AMU. Monocyte^KO^ mice were purchased from Cyagen Biosciences. Diphtheria toxin (DT) inducible system exists in the promoter region of mouse Lyz2, and monocytes are rapidly eliminated once DT is injected.

### 4.4 Western blot

Cell protein was extracted using RIPA lysis buffer (#P0013, Beyotime) plus 1% protease inhibitor (#04693116001, Roche) mixture, and then using BCA kit (#P0010, Beyotime) to detect protein concentration. After separation experiments by SDS–PAGE gel electrophoresis, proteins were transferred to a polyvinylidene‐difluoride membrane. The membrane was sealed with 5% skim milk powder sealing solution at room temperature for 1 h, and then incubated overnight with the primary antibody at 4°C (greater than 10 h). Rinse three times with Phosphate buffer saline (PBS) (PBST) of 0.05% Tween‐20 for 5 min each time, and incubate with horseradish peroxidase secondary antibody at room temperature for 2 h. Then, rinse the membrane three times with PBST for 5 min each time. Finally, the results were observed using the Bio RadChemiDocMP system.

### 4.5 Real‐time polymerase chain reaction

According to the manufacturer's instructions, total RNA was extracted using Trizol reagent (#30237, CWBIO). After concentration determination, 1 µg of RNA was immediately reverse transcribed into cDNA using PrimerScripRT kit (#RR037A, TaKaRa). Quantitative polymerase chain reaction with a total capacity of 20 µL was carried out on ABI 7500 real‐time polymerase chain reaction system (Darmstadt's application biological system in Germany) using TBGreen premix kit (#RR820a, TaKaRa). Relative gene levels were quantified by using GAPDH as a housekeeping gene (2[Ct GAPDH − Ct target gene]).

### 4.6 BrDU proliferation assay

Plant 3 × 10^5^ tumor cells and 2 mL of culture medium in a six‐well plate, culture for 24 h, then add BrDU reagent, continue to culture for 6 h, and then use BrDU cell proliferation kit (BioLegend 370706) to test the cell proliferation ability.

### 4.7 Annexin V–PI apoptosis assay

Plant 5 × 10^5^ tumor cells in a six‐well plate and cultured at 37°C and 5% CO_2_ for 24 h with 2 mL of culture medium per well. Digestion with trypsin to obtain treated and untreated cells (1 × 10^6^) and processed according to the instructions of the Annexin V/PI staining kit (BD Biosciences), followed by analysis using flow cytometry (FACSVerseC6) and FlowJo 7.6.2 software.

### 4.8 Mouse models of peritoneal carcinomatosis

Tumor cells were digested into single cell suspension and resuspended with cold PBS. Six to eight weeks aged male mice were intraperitoneally injected with tumor cells (5 × 10^6^ cells in 100 µL PBS). The mice were sacrificed, and the abdominal tumors were detected and weighed after 2 weeks, or the survival of the mice was observed.

### 4.9 Mouse models of SC tumor

Tumor cells were digested into single cell suspension and resuspended with cold PBS. Six to eight weeks aged male mice were SC injected with tumor cells (5 × 10^6^ cells in 100 µL PBS). The mice were sacrificed, and the SC tumors were detected and weighed after 2 weeks. At the same time, the SC tumor volume of mice was dynamically measured.

### 4.10 Flow cytometry

The spleens were crushed with a 75 µm (F513442, Sangon Biotech) filter screens and dissolved in culture medium. After centrifugation, red blood cell lysis was used to break the red blood for 2 min. Lysis was terminated by adding an equal volume of PBS containing FBS. The obtained cells were resuspended with PBS and passed through 75 µm filter. Then, single cell suspension was obtained.

The obtained tumor tissue was crushed to 1 mm^3^ using ophthalmic scissors, and then the tissues were digested using the digestive solution (1 mg/mL dissolved type IV collagenase, 0.1 mg/mL DNA enzyme, 0.1 mL/mL hyaluronidase) for 1 h at 37°C. Next, the digested solution was filtered and centrifuged. Erythrocytes were lysed similarly as above single cell suspensions of tumor tissue were obtained.

The antibodies included PerCP/Cy5.5 anti‐mouse CD45 antibody (#103132, BioLegend), Alexa Fluor750 anti‐mouse F4/80 antibody (#123116, BioLegend), allophycocyanin (APC) CD206 (MMR) antibody (#141704, BioLegend), phycoerythrin (PE) anti‐mouse CD11c antibody (#117320, BioLegend), fluorescein isothiocyanate (FITC) anti‐mouse CD3 antibody (#100206, BioLegend), APC anti‐mouse CD11b antibody (#101212, BioLegend), FITC anti‐mouse Ly6C antibody (#144011, BioLegend), AF750 anti‐mouse Ly6G antibody (#103122, BioLegend), AF750 anti‐mouse CD4 antibody (#100414, BioLegend), PerCP/Cy5.5 anti‐mouse CD8a antibody (#100734, BioLegend), PE anti‐mouse/human CD3 antibody (#100206, BioLegend), APC anti‐mouse IFNγ antibody (#101212, BioLegend), and AF700 anti‐mouse/human CD45 antibody (#101212, BioLegend).

### 4.11 In vitro phagocytosis assays

Bone marrow‐derived macrophages (BMDMs) were obtained by culturing murine bone marrow with Macrophage Colony stimulating Factor (M‐CSF) for 5−7 days. And then the green fluorescence protein (GFP) labeled tumor cells with four times the number of macrophages were added and incubated for 4−6 h. BMDMs were stained with extracellular antigens using PerCP/Cy5.5 coupled anti‐CD45 and APC coupled anti‐F4/80 antibodies, and then detected using flow cytometry. A total of 40,000 cells were recorded and analyzed in the single cell phylum for each sample. BMDMs containing GFP are believed to be macrophages that engulf tumor cells.

### 4.12 Drug affinity responsive target stability

The experimental methods have been reported in detail in previous studies. In brief, total tumor cell (CT26 or 4T1) proteins were extracted and divided into fractions, followed by incubation with appropriate concentrations of Dimethyl sulfoxide (DMSO) or OS at 37°C for 30 min. Then, different diluted concentrations of protease were added to each group for digestion at room temperature for 10 min and then immediately put on ice to terminate digestion. Finally, the protein was extracted for Western blot experiments.

### 4.13 Statistical analysis

Analyzed data with GraphPad Prism (GraphPad Software, Inc.). Data were expressed as mean ± Standard Error of Mean and analyzed with Student's *t*‐test or Gehan–Breslow–Wilcoxon test. For each parameter of all data presented, n.s. means not significant, ^*^ *p* < 0.05, ^**^ *p* < 0.01, and ^***^ *p* < 0.001.

## AUTHOR CONTRIBUTIONS

*Conceptualization*: W.X., R.S., M.W., and H.M. *Methodology*: R.S., Y.G., N.L., K.Z., and Y.H. *Investigation*: R.S., Y.G., K.Z., Y.H., D.Z., N.L., F.Y., and H.M. *Visualization*: R.S., M.W., and H.M. *Supervision*: H.M. *Writing—original draft*: R.S. and X.P. *Writing—review and editing*: H.M. All authors have read and approved the final manuscript.

## CONFLICT OF INTEREST STATEMENT

The authors declare they have no conflicts of interest.

## ETHICS STATEMENT

Mice: The Animal Care and Use Committee of the Army Medical University (AMU) has reviewed and approved the protocol for the use of animals in this study (no. AMUWEC20226302). Human specimens: Clinical specimens were approved by the AMU Ethics Committee. Written informed consent was obtained from all the patients (no. 2019‐103‐01).

## Supporting information

Supporting information

## Data Availability

All data are available from the corresponding authors upon request.
