# Supplementary material for: Lanosterol synthase deficiency promotes tumor progression by orchestrating PDL1‐dependent tumor immunosuppressive microenvironment
Source: MedComm (2020). 2024 Apr 10;5(4):e528. doi: 10.1002/mco2.528 (PMC11006713; doi:10.1002/mco2.528)
Supplement: Supplementary file 1 — Supporting information [file MCO2-5-e528-s001.docx]

**Lanosterol synthetase deficiency promotes tumor progression by orchestrating PDL1-dependent tumor immunosuppressive microenvironment**

Yuan Gao^1,#^, Kun Zhao^1,#^, Yulan Huang^1,#^, Dapeng Zhang^1^, Na Luo^1^, Feng Yang^4^, Meng Wang^1*^, Rongchen Shi^2,*^, Hongming Miao^1,3,*^

^1^Department of Pathophysiology, College of High Altitude Military Medicine, Third Military Medical University (Army Medical University), Chongqing 400038, China;

^2^Frontier Medical Training Brigade, Third Military Medical University (Army Medical University), Xinjiang, China.

^3^Jinfeng Laboratory, Chongqing, 401329, China.

^4^Department of General Surgery, Xinqiao Hospital, Army Medical University, Chongqing 400037, China.

^#^Theses authors contributed equally to this work.

^*^Corresponding authors

Correspondence to:

Hongming Miao, No. 30 Gaotanyan Street, Shapingba, Chongqing 400038, People’s Republic of China, hongmingmiao@sina.com.

Rongchen Shi, No. 30 Gaotanyan Street, Shapingba, Chongqing 400038, People’s Republic of China, rongchenshitmmu@sina.com.

Meng Wang, No. 30 Gaotanyan Street, Shapingba, Chongqing 400038, People’s Republic of China, mengwangkk23@sina.com.

**Running title:** OS targeting PDL1 promoted tumor progression

**
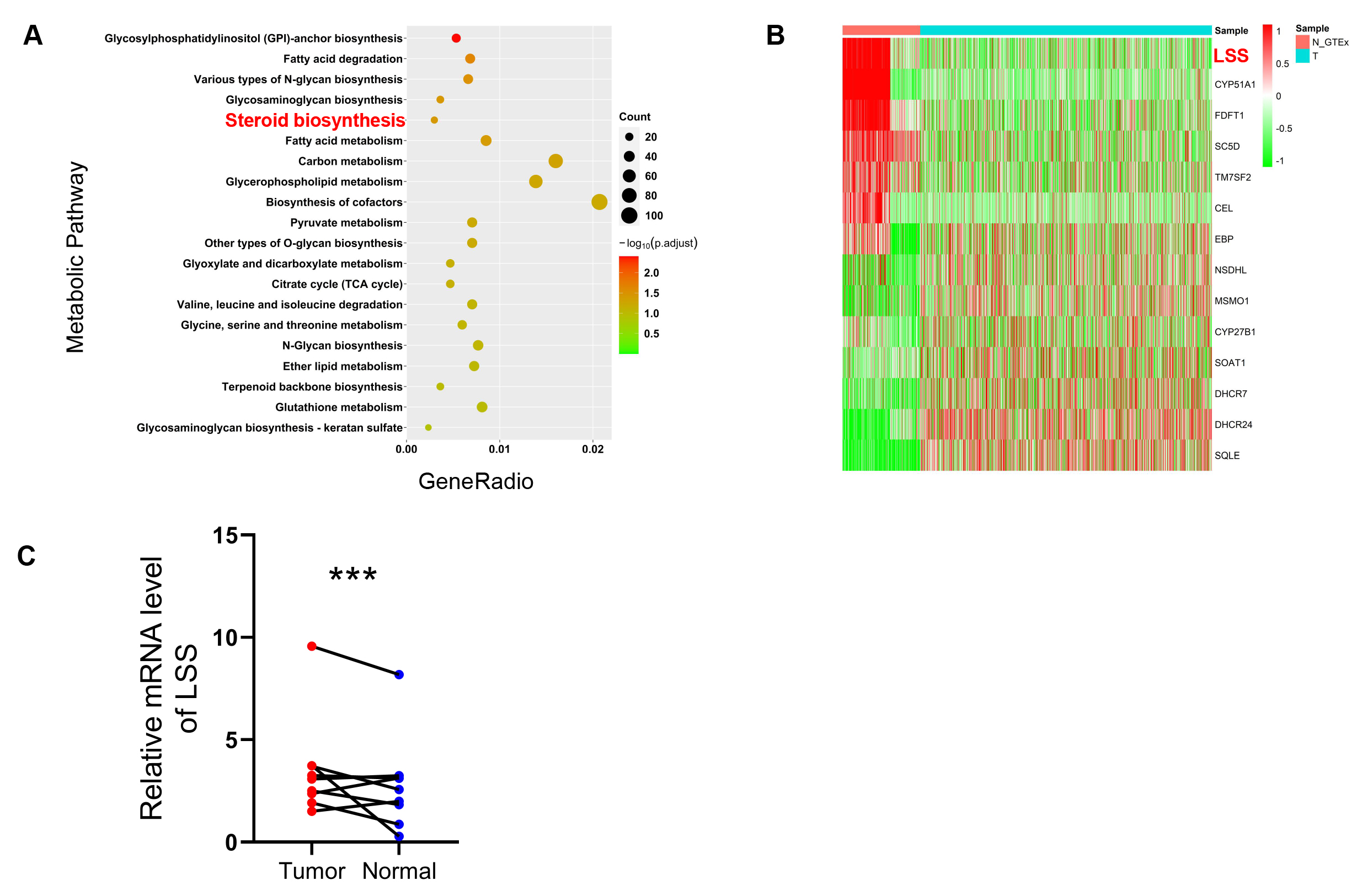
**

**Figure S1 Aberrant expression of LSS in human cancer tissues**

A-B: The RNA sequencing results of adjacent tissues (Pc) and cancer tissues (Ca) of breast cancer patients in TCGA database were analysed. GO pathway enrichment (A) and significantly differentially expressed genes in cholesterol metabolism (B) were performed as indicated.

C. Relative levels of LSS mRNA in cancer and paracancerous tissues of CRC patients. (n=6)

All data represent the means±s.e.ms. (***P < 0.005; A and B are Student’s t-test, C is Paired-sample t test).

**
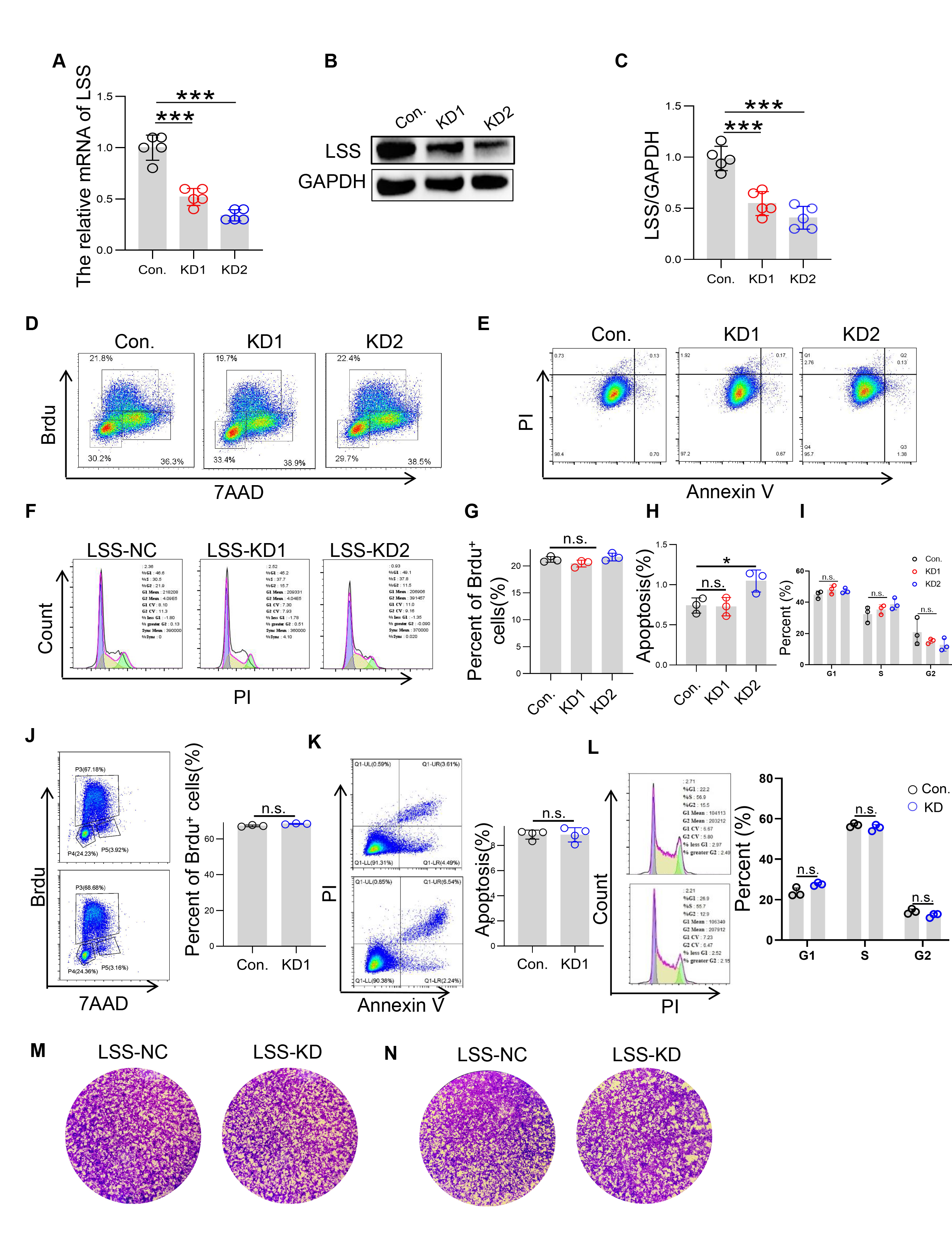
**

**Figure S2 LSS knockdown did not affect the biological activity of tumor cells**

A. The mRNA level of LSS. CT26 were transfected with LSS shRNAs (KD1 and LSS-KD2) or the PLKO vector as a control (Con.), and then the mRNA knockdown effect was detected by real-time PCR. (n=3)

B and C. The protein level of LSS. Immunoblotting assays were used to measure LSS protein levels in Con. or KD cells. (n=3)

D and G. Cell proliferation activity. Con. or KD-CT26 cells were incubated with Brdu for 4 hours and their proliferation activity was detected by Brdu proliferation assay kit. (n=3)

E and H. Cell apoptosis rate. The apoptosis rate of Con. or KD-CT26 cells was detected by Annexin V-APC/PI kit. (n=3)

F and I. The cell cycle of Con. or KD-CT26 cells was detected by flow cytometry. (n=3)

J. Cell proliferation activity of Con. or KD-4T1 cells. (n=3)

K. Cell apoptosis rate of Con. or KD-4T1 cells. (n=3)

L. The cell cycle of Con. or KD-4T1 cells. (n=3)

M. The migration ability of CT26 cells.5×10^4^ LSS-NC or LSS-KDCT26 cells were added to the upper layer of 8μm transwell chambers, and the chambers were obtained after 24 hours of culture. Cells were fixed and stained with crystal violet, and the number of migrating cells was observed by microscopy.
N. The invasion ability of CT26 cells. The transwell chamber was coated with matrigel before the experiment, and then as described in A

All data represent the means±s.e.ms. (**P < 0.01, ***P < 0.005, n.s. not significant; Student’s t-test).


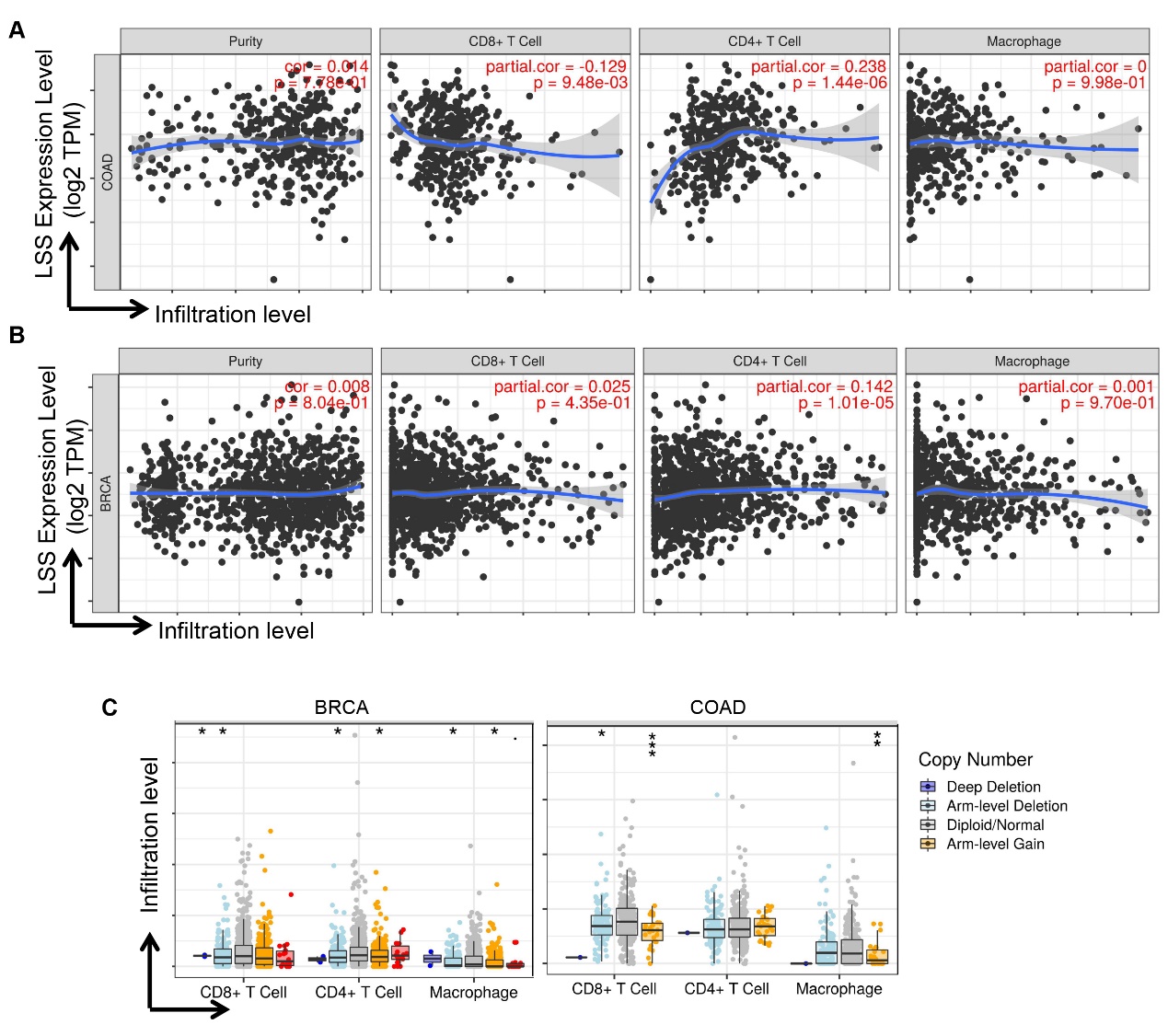


**Figure S3** **LSS is closely related to tumor immune cell infiltration**

A and B. TIMER algorithm was used to study the correlation between LSS genes and tumor-infiltrating immune cells in CRC or breast cancer. Tumor purity is shown in the panels on the left.

C. SCNA module provides the comparison of tumor infiltration levels among CRC or breast cancer with somatic copy number alterations of LSS. SCNAs are defined by GISTIC 2.0., including deep deletion (-2), arm-level deletion (-1), diploid/normal (0), arm-level gain (1), and high amplification (2). Box plots are presented to show the distributions of each immune subset at each copy number status in selected cancer types. The infiltration level for each SCNA category is compared with the normal using a two-sided Wilcoxon rank-sum test.


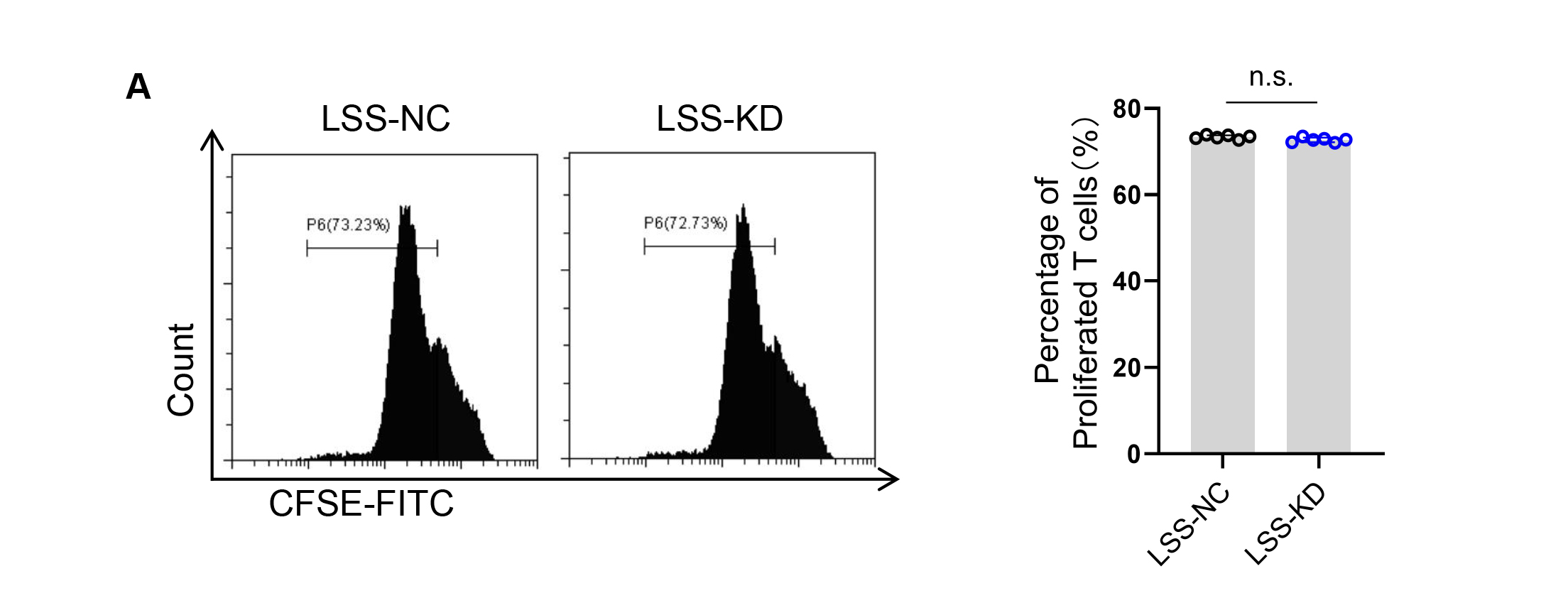


**Figure S4 LSS knockdown did not affect the proliferation of T cells**

1. The single cell suspension was obtained by grinding spleen, and the lymphocytes were separated by lymphocyte separation medium, and then the CD8+ T cells were sorted by CD8+ T cell magnetic beads negative sorting kit. The cells were seeded into CD3-coated dishes and cultured in 1640 medium containing cd28 for 3 days. T cells labeled with CFSE were co-cultured with tumor cells for 24 hours, and the proliferation activity of T cells was detected by flow cytometry.

All data represent the means±s.e.ms. (n.s. not significant; Student’s t-test).


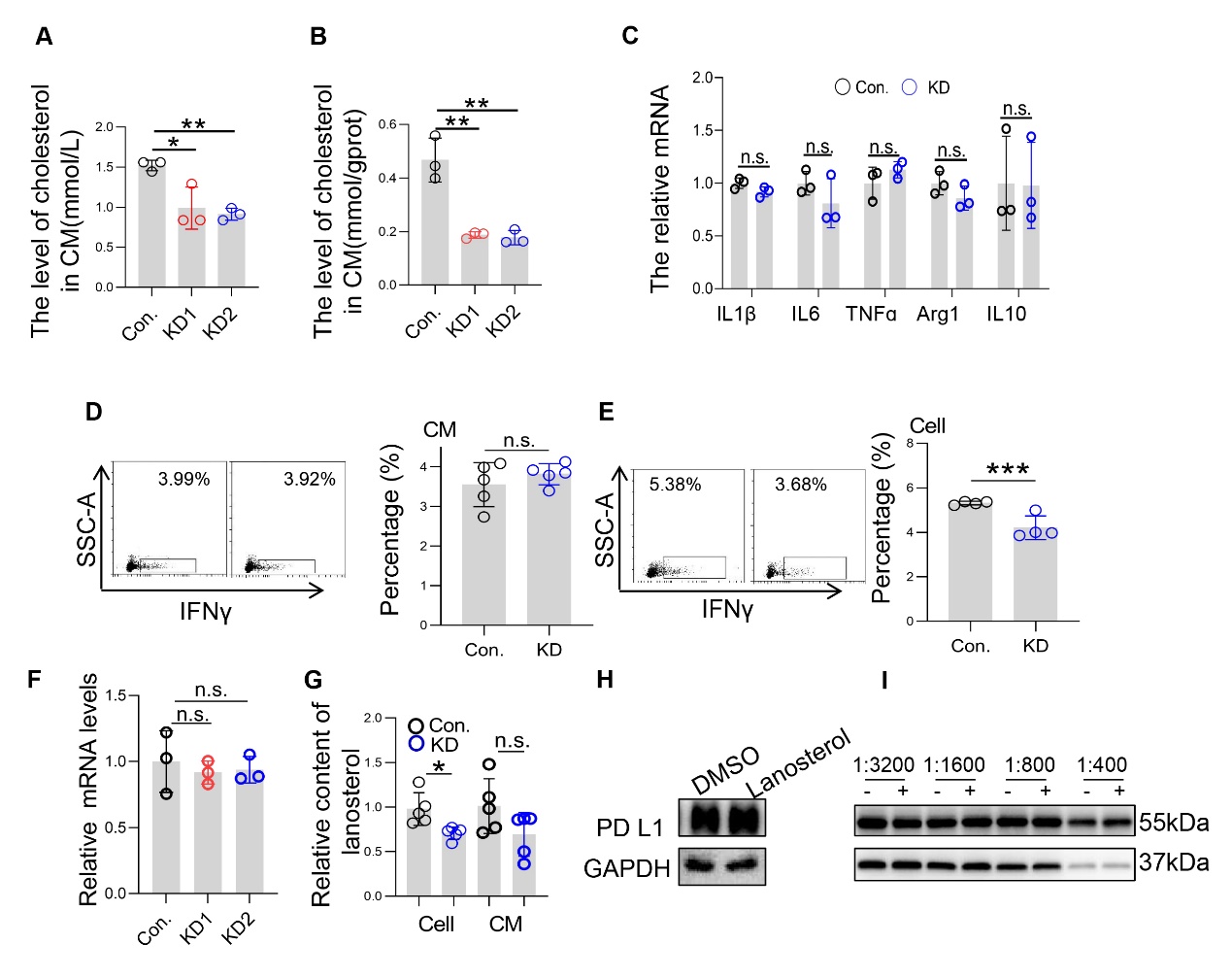


**Figure S5 LSS knockdown promotes tumor progression by enhancing the stability of PDL1 protein**A and B. The level of cholesterol in CM or cells. CM or cells were collected and then the cholesterol content was measured using the total cholesterol detection kit.

C. The relative levels of inflammatory cytokines in macrophages. BMDMs were extracted and induced, and then treated with conditioned medium of Con. or KD-CT26 cells for 24 hours. The relative expression of inflammatory cytokines was detected by real-time PCR.

D. IFNγ expression in T lymphocytes. Splenic leukocytes were treated with the CM of Con. or KD-CT26 cells overnight, and the expression of IFNγ in T lymphocytes was detected by flow cytometry.

E. IFNγ expression in T lymphocytes. Splenic leukocytes were incubated with the Con. or KD-CT26 cells for 4 h, and the expression of IFNγ in T lymphocytes was detected by flow cytometry.

F. The mRNA level of PDL1. LSS-NC or LSS-KD CT26 cells were collected, and real-time PCR was used to measure the mRNA level of PDL1. (n=3)

G. Intracellular (cell) or supernatant(CM) concentrations of lanosterol in Con.- or KD-transfected CT26 cells.

H. The protein level of PDL1. Immunoblotting assays were used to measure PDL1 protein levels in CT26 cells after 36 hours of DMSO or lanosterol treatment. (n=3)

I. The protein level of PDL1 after lanosterol treatment. DARTS experiments are performed as described in the Materials and Methods section.

All data represent the means±s.e.ms (*P < 0.05, **P < 0.01, and n.s. not significant; Student’s t-test).
